# From the Cochrane Library: Interventions for Acne Scars

**DOI:** 10.2196/37060

**Published:** 2022-08-17

**Authors:** Annie L Cao, Torunn E Sivesind, Rania Abdel Hay, Robert P Dellavalle

**Affiliations:** 1 Department of Dermatology University of Colorado Anschutz Medical Campus Aurora, CO United States; 2 Department of Dermatology Faculty of Medicine Cairo University Cairo Egypt

**Keywords:** acne vulgaris, acne scars, fractional laser, injectable filler, chemical peeling, systematic review

Acne vulgaris is a common skin condition that affects both adolescents and adults worldwide and frequently results in acne scars [1]. Atrophic scars are the most common type of acne scars and are caused by a loss of collagen that leads to depressions in the skin surface [2]. Currently, many options exist for acne scar treatment, including lasers, chemical peels, dermabrasion, injectable fillers, needling, subcision, punch excision, and punch elevation. However, providers and patients have few guidelines on how to optimize treatment. Because of the large disease burden and the physical, psychological, and social impact of acne scarring, it is important to provide guidelines for patients and providers on the safest and most effective treatments for this complication.

A 2016 Cochrane study [3] provided a comprehensive review of available treatments and their efficacy for treating facial atrophic acne scars. This review analyzed 24 randomized controlled trials (RCTs) and assessed two primary outcomes: participant-reported scar improvement and serious adverse events that caused withdrawal from the study. Secondary outcomes such as investigator-assessed scar improvement, patient satisfaction, quality of life, participant-reported or investigator-assessed short-term adverse events, and duration of postprocedure downtime were also measured.

Data from some of the included RCTs showed that fractional laser, chemical peeling (with and without skin needling), and injectable fillers were more effective than comparator treatments. Many studies that compared other treatment modalities to each other or to placebo concluded no significant difference in either participant-reported or investigator-assessed scar improvement. Tables 1 and 2 summarize the treatment comparisons of the 24 included RCTs.

This review [3] found moderate support for the use of injectable fillers in acne scar treatment and limited support for lasers, chemical peeling, radiofrequency, and skin needling. The authors could not recommend one treatment modality over another due to insufficient evidence supporting any particular treatment. The included studies were generally underpowered and had a high risk for bias due to lack of blinding and participants’ expectations of treatment influencing improvement ratings. Assessment of acne scar treatment efficacy poses challenges secondary to differences in study parameters across studies, variable subjective improvement rating scales, and lack of long-term follow-up of scar improvement. Additional RCTs with larger study populations, sham and/or placebo trials, and standardized outcomes and improvement ratings are necessary to determine the efficacy of treatment [3].

Results of clinical trials published subsequent to this review [3] provide further insight. A double-blind, parallel, multicenter RCT [4] compared the effects of polymethylmethacrylate (PMMA) microspheres in collagen (ArteFill) injections to placebo (saline injections) as a treatment for acne scarring and reported treatment success in 64% of treated participants vs 33% of control participants after 6 months (*P*=.0005). Another multicenter, randomized, prospective study [5] compared combination microneedling with PMMA-collagen gel filler injections vs microneedling alone, and found the combination group had significantly improved acne scar scores at 24 weeks post treatment compared to the microneedling-alone group (*P*=.0136). These studies further support the efficacy of injectable fillers for treating acne scars, though additional research with long-term follow-up is warranted to assess the durability of outcomes.

**Table 1:** Comparison of interventions for acne scars.^a^

Comparison^a^	Study details	Scar improvement	Adverse events	Quality of evidence	Risk of bias
Nonfractional nonablative (NFNA) laser vs placebo/no treatment	Frequency-doubled 532-nm Nd:YAG (neodymium:yttrium-aluminium-garnet) laser; within-individual study	Participant reported (PR): 53.6% improvement in acne scarring (range: 10%-90%); no data for untreated	None reported	Not assessed	High risk of detection bias
*Fractional laser (FL) vs NFNA laser*	CO_2_ FL vs Q-Switched 1064-nm Nd:YAG laser; parallel-group study	PR: 12/32 (FL) vs 3/32 (NFNA laser) participants reported >50% improvement in scars at 6 months (risk ratio [RR] 4.00, 95% CI 1.25-12.84)	Transient posttreatment burning sensation in the NFNA group; postinflammatory hyperpigmentation (PIH) reported in 16/64 subjects	Very low-quality evidence	Unclear risk of detection bias
FL vs placebo/no treatment	1540-nm Er:Glass FL; within-individual study	PR: 8/10 patients reported improved acne scars after 12 weeks; no data for untreated	Immediate pain and transient erythema post treatment	Not assessed	High risk of detection bias
FL vs placebo/no treatment	CO_2_ FL; within-individual study	PR: 12/12 subjects reported mild to moderate improvement in scars after 6 months; no data for untreated side	Mild to moderate pain, erythema, and wound formation	Not assessed	High risk of detection bias
FL vs radiofrequency (RF)	1550-nm Er:Glass FL vs fractional RF; parallel-group study	PR: 7/20 (FL) vs 9/20 (RF) participants reported >50% improvement in acne scarring at <24 weeks post treatment (RR 0.78, 95% CI 0.36-1.68)	Pain with FL greater than with RF; both groups reported erythema and edema; PIH in the FL group only	Very low-quality evidence	High risk of detection bias
FL vs RF	1550-nm Er:Glass laser vs fractional bipolar RF; within-individual	PR: mean improvement grade in acne scars after treatment; fractional laser (2.89, SD 0.57) vs RF (2.74, SD 0.73)	1/20 participants withdrew due to prolonged dyspigmentation negatively affecting quality of life	Not assessed	Unclear risk of detection bias
FL vs RF	10,600-nm CO_2_ FL vs fractional microplasma RF; within-individual	Investigator assessed (IA)^c^: acne scar improvement in FL (59.2%) vs RF (56.4%) (*P*=.93)	Posttherapy erythema, scaling, and PIH were more significant on the FL side	Not assessed	Not assessed
*FL vs combined FL with any active intervention*	10,600-nm CO_2_ FL alone vs same laser plus punch elevation; within-individual	IA^c^: 26/42 (FL) vs 31/42 (FL with punch elevation) investigators reported >50% acne scar improvement at <24 weeks (RR 1.45; *P*=.02)	Transient erythema, crusting, transitory burning after treatment, and mild PIH occurred with both interventions	Not assessed	Not assessed
FL vs combined FL with any active intervention	CO_2_ FL with saline vs CO_2_ FL with autologous platelet-rich plasma (PRP); within-individual	IA^c^: mean degree of clinical improvement for FL (2.3, SD 0.5) vs FL with PRP (2.7, SD 0.7)	Posttreatment crusting and edema lasted significantly longer on the FL-alone side than on the combined treatment side	Not assessed	Not assessed
FL vs chemical peeling (CP)	1550-nm Er:Glass FL vs chemical reconstruction of skin scars CP method; within-individual	IA^c^: average improvement grades after <24 weeks: FL (2.51) vs CP (2.44)	1/20 participants left the trial due to minor discomfort with treatment from pain and redness	Not assessed	Not assessed
FL vs combined CP with needling	Nonablative 1540-nm Er:Glass FL vs CP with trichloroacetic acid (TCA) 20% with skin needling; parallel-group	PR: 9/13 (FL) vs 9/13 (combined CP with needling) participants reported >50% acne scar improvement after 12 months (RR 1.00, 95% CI 0.60-1.67)	Pain, transient edema, and erythema were reported in both groups	Very low-quality evidence	High risk of detection bias
*CP vs placebo/no treatment*	Glycolic acid peels (at different concentrations) vs 15% glycolic acid cream vs placebo cream; parallel-group study^d^	IA^c^: significantly better response in the CP group vs placebo (*P*<.05)	CP group: 7 participants withdrew (intolerance to high concentrations, longer contact times of peeling agent); RR 5.45, 95% CI 0.33-90.14	Very low-quality evidence	High risk of attrition bias

^a^Studies did not stratify patients based on acne severity (mild, moderate, severe), which may affect response to scar treatment.

^b^Italicized studies indicate statistically significant study results.

^c^Patient-reported scar improvement was not assessed in this study; investigator-reported scar improvement results were included.

^d^Both treatment arms (glycolic acid peels and glycolic acid creams) were combined into 1 treatment comparison group for analysis.

**Table 2:** Comparison of interventions for acne scars (continued).^a^

Comparison^a^	Study details	Scar improvement	Adverse events	Quality of evidence	Risk of bias
Chemical peeling (CP) vs combined CP plus any active intervention	Deep peeling with oil phenol in a 60% concentration formula nonhydroalcoholic solution vs trichloroacetic acid (TCA) 20% with skin needling; parallel-group study	Participant reported (PR): 10/10 (CP) vs 8/10 (CP with needling) participants reported >50% acne scar improvement after 8 months (RR 1.24, 95% CI 0.87-1.75)	All participants reported pain and transient erythema in both groups	Very low-quality evidence	High risk of detection bias
CP vs needling	100% TCA chemical reconstruction of skin scars (CROSS) vs skin needling using dermaroller; parallel-group study	PR: 9/12 (TCA CROSS) vs 10/15 (skin needling) participants reported >50% acne scar improvement at 1 month (RR 1.13, 95% CI 0.69-1.83)	All participants reported pain and transient erythema in both groups; 6/12 participants in the peeling group experienced postinflammatory hyperpigmentation (PIH)	Very low-quality evidence	High risk of detection and attrition bias
*Needling vs placebo/no treatment*	Needling vs topical anesthetic cream; within-individual study	PR: 41% mean improvement in acne scars on the treated side	All participants reported pain, and transient erythema and edema were seen in all participants	Not assessed	Not assessed
*Injectable fillers vs placebo/no treatment*	Polymethylmethacrylate suspended in bovine collagen vs saline injections; parallel-group study	PR: 77% (injectable filler) vs 42% (placebo) of participants reported improved acne scarring (RR 1.84, 95% CI 1.31-2.59; *P*<.05)	Injection site pain, injection site tenderness, swelling, erythema, bruising, pain, itching, lumps or bumps, and discoloration	Moderate-quality evidence	Low risk of detection bias
*Injectable fillers vs placebo/no treatment*	Autologous fibroblasts vs vehicle control; within-individual study	PR: 43% of treated sides showed ≥2-point acne scar improvement compared with 18% of the vehicle-control treated side (*P*<.001)	Participants in both groups reported mild to moderate erythema	Not assessed	Low risk of detection bias
Injectable fillers vs subcision	Injectable filler with natural-source porcine collagen vs 18-gauge Nokor subcision needle; within-individual study	PR: 3.5 (injectable filler) vs 3.9 (subcision) global improvement rate (*P*=.12)	Higher severity of bruising reported with subcision vs fillers	Not assessed	High risk of detection bias
Microdermabrasion (MDA) + aminolevulinic acid (ALA)–photodynamic therapy (PDT) vs MDA + placebo-PDT	417-nm blue light therapy plus MDA with 20% δ-ALA or vehicle solution	Investigator assessed (IA)^c^: 80% of participants showed acne scar improvement on the MDA + ALA-PDT side vs the MDA + vehicle-PDT side	None reported	Not assessed	Not assessed
Fractional laser (FL) vs FL	Er:YAG FL vs CO_2_ FL laser; within-individual	PR: 70% (Er:YAG) vs 60% (CO_2_) of laser sites were rated as showing >50% improvement in acne scarring (*P*=.47)	Participants reported erythema, edema, superficial crusting, and PIH	Not assessed	Not assessed
Photothermolysis vs FL	Nonablative 1550-nm erbium-doped fractional photothermolysis system (FPS) vs 10,600-nm CO_2_ FL system; within-individual	IA^c^: mean grade of improvement for FPS (2.0, SD 0.5) vs FS (2.5, SD 0.8) (*P*=.158)	Mean pain scores were significantly lower for FPS than with FL; side effects included crusting, scaling, redness, fluid retention, and hyperpigmentation	Not assessed	Not assessed
Pulsed dye laser (PDL) vs long-pulsed laser	Nonfractional nonablative (NFNA) PDL vs 1064-nm long-pulsed Nd:YAG (neodymium:yttrium-aluminium-garnet) laser; within-individual	IA^c^: acne scores improved by 18.3% (PDL) and 18.7% (Nd:YAG); no statistically significant difference between treatments	Reported adverse events included transient pain, erythema, and edema in treated areas	Not assessed	Not assessed
Long-pulsed Nd-YAG laser vs diode laser	NFNA 1320-nm long-pulsed Nd-YAG laser vs NFNA 1450-nm diode laser; within-individual	IA^c^: higher average clinical scores on 1450-nm diode laser–treated face side than on Nd-YAG laser–treated face side	All participants experienced posttreatment erythema, and some had PIH and discomfort with treatment	Not assessed	Not assessed
Long-pulsed Nd-YAG laser vs combined laser	Long-pulsed Nd:YAG laser vs combined 585/1064-nm laser; within-individual	IA^c^: acne scores improved by 27% (Nd:YAG) and 32.3% (585/1064-nm laser); no statistically significant difference	Reported adverse events included transient pain, erythema, and edema in both treated areas	Not assessed	Not assessed

^a^Studies did not stratify patients based on acne severity (mild, moderate, severe), which may affect response to scar treatment.

^b^Italicized studies indicate statistically significant study results.

^c^Patient-reported scar improvement was not assessed in this study; investigator-reported scar improvement results were included.

## Abbreviations

PMMA: polymethylmethacrylate
RCT: randomized controlled trial
